# Unveiling Novel ERCC1–XPF Complex Inhibitors: Bridging the Gap from In Silico Exploration to Experimental Design

**DOI:** 10.3390/ijms25021246

**Published:** 2024-01-19

**Authors:** Rita Manguinhas, Patrícia A. Serra, Rita B. Soares, Rafael Rosell, Nuno Gil, Nuno G. Oliveira, Rita C. Guedes

**Affiliations:** 1 Research Institute for Medicines (iMed.ULisboa), Faculty of Pharmacy, Universidade de Lisboa, 1649-003 Lisboa, Portugal; rmanguinhas@campus.ul.pt (R.M.); pfserra@ff.ulisboa.pt (P.A.S.); soares.rita@edu.ulisboa.pt (R.B.S.); 2 Lung Unit, Champalimaud Clinical Centre (CCC), Champalimaud Foundation, 1400-038 Lisboa, Portugal; nuno.gil@fundacaochampalimaud.pt; 3 Egas Moniz Interdisciplinary Research Center, Instituto Universitário Egas Moniz, 2829-511 Caparica, Portugal; 4 Dr. Rosell Oncology Institute, 08028 Barcelona, Spain; rrosell@iconcologia.net; 5 Catalan Institute of Oncology, 08916 Barcelona, Spain

**Keywords:** DNA repair pathways, ERCC1–XPF complex, inhibitors, structure-based virtual screening, structural analysis, cisplatin, NSCLC

## Abstract

Modifications in DNA repair pathways are recognized as prognostic markers and potential therapeutic targets in various cancers, including non-small cell lung cancer (NSCLC). Overexpression of ERCC1 correlates with poorer prognosis and response to platinum-based chemotherapy. As a result, there is a pressing need to discover new inhibitors of the ERCC1–XPF complex that can potentiate the efficacy of cisplatin in NSCLC. In this study, we developed a structure-based virtual screening strategy targeting the inhibition of ERCC1 and XPF interaction. Analysis of crystal structures and a library of small molecules known to act against the complex highlighted the pivotal role of Phe293 (ERCC1) in maintaining complex stability. This residue was chosen as the primary binding site for virtual screening. Using an optimized docking protocol, we screened compounds from various databases, ultimately identifying more than one hundred potential inhibitors. Their capability to amplify cisplatin-induced cytotoxicity was assessed in NSCLC H1299 cells, which exhibited the highest ERCC1 expression of all the cell lines tested. Of these, 22 compounds emerged as promising enhancers of cisplatin efficacy. Our results underscore the value of pinpointing crucial molecular characteristics in the pursuit of novel modulators of the ERCC1–XPF interaction, which could be combined with cisplatin to treat NSCLC more effectively.

## 1. Introduction

Non-small cell lung cancer (NSCLC), the predominant subtype of lung cancer (LC), remains a significant global health challenge, contributing to a substantial proportion of cancer-related deaths [1,2]. Despite significant advances in treatment strategies, many NSCLC patients continue to face reduced survival rates due to factors such as late diagnosis, metastatic progression, and both intrinsic and acquired resistance to therapies [3,4].

Cells undergo genomic instability at multiple stages of their life cycle. As a result of this instability, DNA lesions, either from endogenous or exogenous sources, are constantly formed. To preserve genomic integrity, cells have evolved a series of DNA repair pathways [5,6]. While these pathways are essential for cellular health, cancer cells can exploit them for their benefit [7,8,9]. An increased expression of several genes involved in DNA repair mechanisms has been identified as biomarkers of cancer progression and has been associated with adverse clinical outcomes [10,11,12,13,14]. Moreover, these repair pathways can also act as a double-edged sword for tumoral cells by providing a survival advantage when confronted with DNA-damaging agents, such as platinum-based drugs like cisplatin. Resistance to cisplatin has been associated with an increased DNA repair response in tumors, including NSCLC [15,16,17,18,19]. Particularly, the overexpression of the excision repair cross-complementation group 1/xeroderma pigmentosum complementation group F (ERCC1–XPF) in the Nucleotide Excision Repair (NER) pathway has been linked to the augmented repair of cisplatin-mediated DNA damage [12,16,20]. Consequently, recent research has shifted towards targeting DNA repair pathways to enhance the efficacy of existing cancer treatments by sensitizing tumor cells to these therapeutic agents and potentially circumvent resistance [21]. This paradigm shift has given rise to an emerging cancer research field focused on the synergistic use of DNA repair inhibitors with standard chemo/radiotherapy or as standalone treatments employing synthetic lethality [22,23,24,25,26]. This approach, termed “DNA repair targeted therapy”, has yielded promising drug candidates, including poly(ADP-ribose) polymerase (PARP) inhibitors, some of which are in clinical trials or have received regulatory approval [27].

In the context of NSCLC, ERCC1 overexpression has been extensively investigated. Elevated ERCC1 expression levels have pivotal implications in prognosis, therapeutic responses, and personalized treatment strategies [12,20,28,29]. Given that ERCC1 overexpression is tied to suboptimal outcomes with cisplatin regimens, this protein emerges as a promising target for drug development in NSCLC [30,31]. The ERCC1 forms a heterodimer with XPF, forming the ERCC1−XPF complex, a critical component for DNA repair within the NER pathway [30]. While ERCC1 modulates various DNA and protein interactions, the endonuclease activity is attributed to XPF, which also harbors a non-functional helicase-like motif implicated in protein−protein interactions and DNA binding [31,32]. Prior computational studies aimed to identify potential inhibitors of this complex, shedding light on important structural features vital for its function. Initial attempts at inhibiting this complex focused on disrupting the interaction between xeroderma pigmentosum group A (XPA) and ERCC1, with XPA playing a role in recruiting ERCC1–XPF to DNA-damaged sites within the NER pathway [33,34]. While certain inhibitors showed promising results, they lacked specificity for other processes in which ERCC1–XPF, but not XPA, is involved, such as interstrand cross-link repair (ICL repair) and DNA double-strand break (DSB) repair [35]. Other authors have targeted the interactions between XPF and DNA and between XPF and ERCC1; however, many of these molecules have issues related to off-target cytotoxicity, poor pharmacokinetics, and reduced potency [36,37,38,39,40,41,42,43,44,45].

In this study, we introduce an alternative approach for identifying novel and more efficient ERCC1–XPF inhibitors for combination therapy with cisplatin in NSCLC treatment. We implemented a structure-based virtual screening (SBVS) strategy, emphasizing the disruption of ERCC1 and XPF interactions. Using crystallographic data on ERCC1–XPF, we characterized the interface domain. A curated library of small molecules with reported activity for the ERCC1–XPF complex was extracted from ChEMBL to validate the SBVS protocol. Molecular docking and virtual screening calculations were performed and subsequently used to screen and rank approximately 2 million compounds from various databases. Compounds were selected based on their score, molecular weight, lipophilicity, ligand efficiency, appropriate pocket fit, and interactions with nearby residues. These selected inhibitors underwent validation in cell-based in vitro assays with a NSCLC cell line with higher ERCC1 expression and in combination with cisplatin. Altogether, this study provides a proof of concept for potential ERCC1–XPF inhibitors, introducing new chemotypes for use in combination therapy with cisplatin for NSCLC treatment.

## 2. Results and Discussion

### 2.1. Computational Studies on the ERCC1–XPF Protein Complex

The ERCC1–XPF heterodimer serves as a structure-specific 5′–3′endonuclease, essential for DNA damage repair via the NER pathway. While ERCC1 is catalytically inactive, it plays a vital role in modulating DNA–protein and protein–protein interactions. The endonuclease activity, on the other hand, is provided by XPF. Additionally, XPF features an inactive helicase-like motif, which is believed to participate in protein−protein interactions and DNA binding.

To deepen our understanding, we adopted a structure-based virtual screening (SBVS) strategy. This strategy encompassed a comprehensive structural and physicochemical analysis and characterization of the ERCC1–XPF complex. We subsequently refined a library of small molecules known to exhibit activity against the complex. This approach is critical for gathering crucial information on structural properties, binding regions, flexibility, key binding pocket interactions, and the dynamics of ligand–receptor interactions. This knowledge is the cornerstone for the cutting-edge development of new drugs with enhanced properties.

#### 2.1.1. Structural Analysis of the Protein Complex

Understanding structural properties, binding regions, flexibility, key interactions within binding pockets, and the dynamics of ligand–receptor interactions are essential for drug development. To achieve a comprehensive structural analysis and characterization of the ERCC1–XPF complex, we explored available three-dimensional (3D) structures. At the beginning of our investigation, eight 3D structures of the ERCC1–XPF complex had been published and made available in the Protein Data Bank (PDB) (Table 1) [46]. It is worth noting that none of these structures had bound a small-molecule inhibitor. The first partial structure was published in 2005, and only recently has a complete cryo-EM structure of the complex been made available [47,48,49,50,51,52]. These structures pertain to the catalytic domain, shedding light on key residues within the binding region and structural aspects that can guide the optimal selection for subsequent virtual screening campaigns.

**Figure 1:**
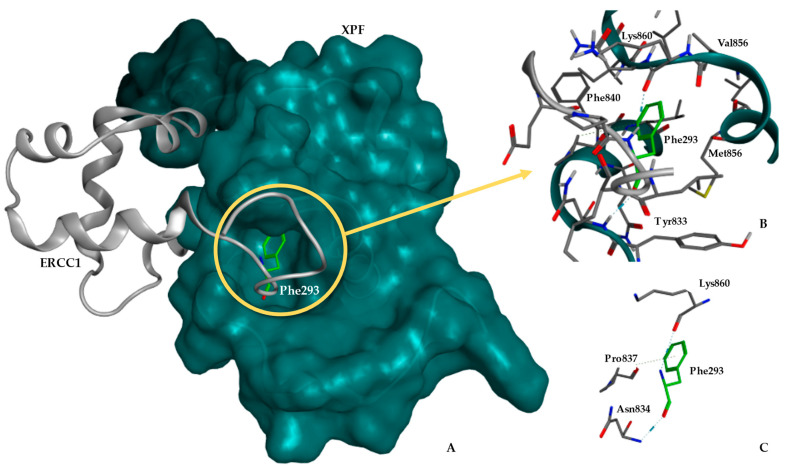
ERCC1–XPF complex structure with emphasis on the binding site and its interactions: (**A**) The ERCC1–XPF complex is depicted with emphasis on the XPF pocket enveloping Phe293 from ERCC1, based on the 1Z00 PDB structure. The molecular surface, tinted in aqua, illustrates the surface on the C-terminal region of the XPF protein. This visualization showcases how the ERCC1 protein is complexed with the XPF protein; (**B**) A close-up of the pocket region reveals Phe293 (highlighted in green) forming a hydrogen bond with Lys860 of XPF and participating in aromatic interactions with both Pro837 and Asn834; (**C**) An in-depth perspective of the XPF residues interacting with Phe293 is provided.

In the first analysis, we aligned and superimposed the retrieved 3D structures of ERCC1 and XPF proteins, as shown in Table 1 (PDBIDs: 1Z00, 2A1J, 6SXA, and 6SXB). These proteins contain double helix–hairpin–helix (HhH2) motifs, crucial for dimerization. The superimposition unveiled significant similarity, particularly in the vicinity of the putative binding pocket region. This region is defined around Phe293 of ERCC1, as identified using Molecular Operation Environment (MOE) software version v.2020.0901 [53]. The root mean square deviations (RMSDs) ranged from 1.30 Å for ERCC1 to 1.60 Å for XPF. A careful analysis of the complex highlighted that the pocket enveloping Phe293 of ERCC1 is highly conserved, suggesting its potential as a key site for inhibiting the complex’s activity. The aromatic ring of Phe293 fits within the hydrophobic cavity of the XPF protein, defining a pocket with a large contact surface area of 280 Å^2^, as calculated using MOE (Figure 1).

Our structural analysis unveils clear interactions between the two proteins within the complex. The ERCC1 backbone forms crucial interactions with XPF, establishing three hydrogen bonds that anchor the side chain of Phe293 within the pocket. As depicted in Figure 1, the interactions involving Phe293 of ERCC1, and residues Asn834, Pro837, and Lys860 of XPF, play a significant influence in stabilizing the complex. Figure 1 further illustrates the interaction between the C-terminal regions of ERCC1 and XPF, leading to heterodimer formation, facilitated by the HhH2 motifs present in both proteins [37]. The activity domain of ERCC1 adopts its functional conformation only alongside XPF, serving as a foundation for the proper folding of ERCC1. When isolated, both structures are prone to degradation, lacking structural stabilization [38,41,44,51,54]. Notably, the deletion of the single residue Phe293 from ERCC1 is sufficient to stop the dimerization process between ERCC1 and XPF. This is anticipated given its strategic position within the binding pocket region [38]. The residue Phe840 in XPF is also pivotal for nonbonded interactions within the complex, as its aromatic ring interacts with several ERCC1 residues (see Figure 1B). Additional important features are evident in the interface region, including the pocket’s encapsulation by the side chains of Tyr833 from XPF and Leu294 from ERCC1, both interacting with Phe293, located within the pocket.

Among the four structures analyzed, the 1Z00 PDB structure contains the highest number of residues in the pocket’s critical region. This aspect has been previously emphasized by several authors in the context of designing small molecule inhibitors [39,40,42,55].

**Figure 2:**
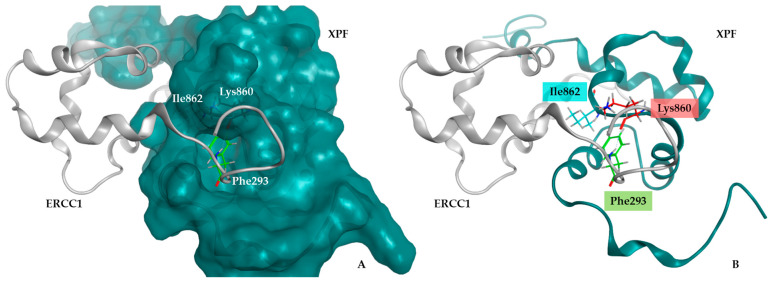
(**A**) Depiction of the ERCC1–XPF complex highlighting the XPF pocket formed around Phe293 from ERCC1. The relative positions of residues used to validate the molecular docking protocol within the XPF pocket are also evident; (**B**) A close-up view of the pocket region showcases Phe293 from ERCC1 (green) and the two residues from XPF: Leu860 (red) and Ile862 (blue). These residues delineate the upper bounds of the pocket.

Our pocket analysis indicates that the residues Ile862 and Lys860 from XPF face the pocket, suggesting potential guiding capabilities for small molecules. Specifically, Ile862 was selected due to its location on the inner side of the pocket, implying its potential to guide molecules into the pocket. Consequently, these residues were selected and evaluated as potential central residues for defining the center of the docking box (binding site) in subsequent calculations (see Figure 2A,B). The generated binding pocket predominantly possesses hydrophobic properties, characterized by its significant depth. Yet, at the frontal face where the interface is more exposed, a predominantly hydrophilic region emerges, as inferred from the protein hydrophilic/lipophilic surface calculated using MOE.

#### 2.1.2. Assembly of an ERCC1–XPF Small Molecule Inhibitors Set

To select and validate the docking protocol, we amassed a collection of compounds known for their biological activity against the target, sourced from the ChEMBL28 database [56]. The literature review revealed numerous studies that have employed the compound designated as F06 as an initial reference. Despite F06 having a recognized yet suboptimal pharmacokinetic and physicochemical profile, it has been frequently utilized as a starting point in various studies due to its ability to disrupt the interaction between ERCC1 and XPF. Notably, F06 interacts within the pocket defined in this study, forming a complex with XPF. Recognizing its inherent limitations, researchers have synthesized analogous compounds to enhance their properties [36,38,41,44]. The inhibitor set showcases activities from as low as 330 nM to those deemed inactive (activities above 100 μM).

**Figure 3:**
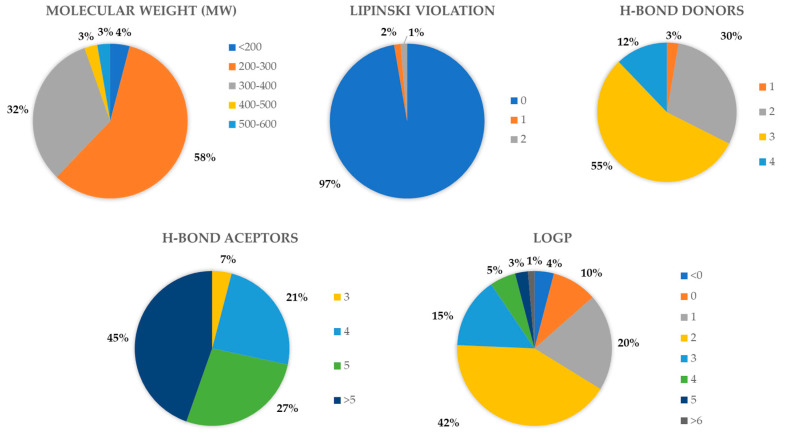
Graphical representation of the distribution of the molecular descriptors of the molecules retrieved from ChEMBL with activities up to 30 µM. The descriptors assessed include molecular weight (MW), Lipinski violations, H-bond donors, H-bond acceptors, and LogP. A total of 74 molecules were analyzed.

A pool of 84 molecules, known for their activity against ERCC1–XPF, was gathered and subjected to an exhaustive analysis. This centered on diverse properties and descriptors, including molecular weight, H-bond acceptors, H-bond donors, LogP, rotatable bonds, heavy atoms, and Lipinski violations, among others. Molecular descriptors were computed using FAF-Drugs4 [57] and MOE v.2020.0901, which spanned both conventional and pharmacophore-based descriptors aligned with drug-likeness rules, ideal drug attributes, and medicinal chemistry properties. These molecules exhibit structural diversity, ranging from molecular masses of under 200 g/mol to 600 g/mol, accompanied by a broad spectrum of LogP values, suggesting varying lipophilicity levels (refer to Figure 3).

The compound set was employed to develop and validate the docking protocol. The analysis of molecules displaying activity up to 30 µM revealed that the molecular weight primarily ranged between 200 and 400 g/mol, featuring 2 to 3 H-bond donors and 4 or more H-bond acceptors. The LogP values were generally between 1 and 3 with no violations of Lipinski’s rule of five. These data are crucial for selecting compounds after virtual screening.

#### 2.1.3. Optimization of Docking Protocol and Virtual Screening Campaign

To ascertain and validate an optimal docking protocol for subsequent virtual screening campaigns in extensive molecular databases, a series of docking simulations were conducted. Four widely recognized scoring functions available in the GOLD v5.07 software suite (ChemPLP, GoldScore, ChemScore, and ASP) were assessed. The small molecules from the ChEMBL dataset underwent preprocessing, entailing the retrieval of corresponding SMILES notations, conversion to 3D structures, and preparation under physiological conditions (pH 7.4 and 300 K) using MOE v.2020.0901 [53]. An energy minimization step using the semi-empirical PM7 method ensured an energetically favorable starting conformation.

Each small molecule was then docked into the four complex crystal structures, with three independent systems defined as receptors: complex, XPF, and ERCC1 alone. This used all available scoring functions. The evaluation examined the scoring functions and systems for scoring accuracy, ranking capability, and ligand efficiency. The docking site (center) was defined using a specific set of residues as a reference (performed individually for each residue) within a 15 Å radius. Furthermore, the binding region for the most energetically favorable small molecule conformations was determined to encompass the interaction zone within the complex, specifically the region close to the Phe293 residue.

Upon completion of the docking simulations, the results were analyzed to establish correlations between each molecule’s score and its documented inhibitory activity. The primary goal was to enhance the protocol’s ability to distinguish highly active compounds and assign a more accurate score. Using the complex as a system showed poorer performance as the molecules could not access the binding region. Docking into individual proteins, the XPF system displayed improved occupancy. Evaluating all molecular docking results for the 84 ChEMBL molecules, the optimal and most reproducible parameters identified and selected involved the GOLD v5.07 software, the ChemPLP scoring function (with 500 GA runs), docking at the Lys860 residue in XPF, and employing the 1Z00 crystallographic structure (presented the best placement of the molecules into the pocket with the best correlation between score and inhibitory activity comparing all systems).

Following the established docking protocol, the virtual screening process was initiated for the NCI (284,176 compounds), ChemBridge (1,008,227 compounds), and DrugBank (524 compounds) databases, totaling 1,292,927 molecules. These databases of compounds offer a plethora of diverse chemical structures essential for virtual screening, increasing the chances of identifying novel and potent lead compounds. Their diversity ensures a comprehensive exploration of chemical space during virtual screening (various functional groups, scaffolds, and stereochemical configurations).

**Figure 4:**
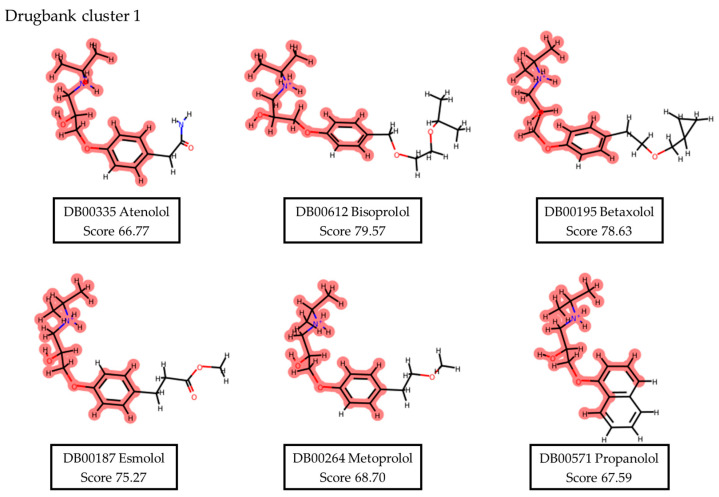
Representation of cluster 1 derived from the DrugBank results dataset proposed from the visual inspection of a total of 48 molecules. Parts of the molecules highlighted in red represent the similar scaffold that defines each cluster.

Compounds from the virtual screening (for each of the databases) were prioritized based on score, ligand efficiency, optimal pocket complementarity (via visual inspection), and interactions with neighboring residues. Results were filtered focusing on score, molecular weight, ligand efficiency, LogP, and Pan-assay interference compounds (PAINS) identification. From the top 5000 molecules’ scored compounds, those exceeding a score of 90 underwent further descriptor-based filtering, visual inspection, and clustering of structurally similar scaffolds using Murcko Scaffold decomposition in RDKit [58]. The similarity was assessed using the Tanimoto coefficient computed over Morgan fingerprints, a method also implemented in RDKit. This extensive evaluation refined the molecules’ selection for this challenging target, diversifying the scaffold assortment within the chosen set of molecules (see Figure 4).

**Figure 5:**
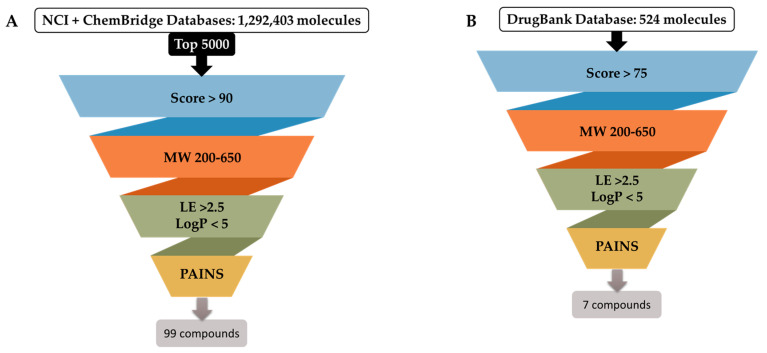
Schematic representation of the filtering criteria applied for compound selection from the selected databases. A total of 1,292,927 molecules from the three databases were subjected to docking (individually): (**A**) From both the NCI and ChemBridge databases, 1,292,403 molecules were scored to find the top 5000 molecules. They were then selected for further filtration using the specified criteria and, consequently, 99 compounds were selected for acquisition (64 from ChemBridge and 35 from NCI databases). (**B**) From the DrugBank database, all 524 molecules were scored and rated using the specified criteria resulting in 7 compounds proposed for acquisition.

The DrugBank database’s compounds (524 approved drugs) underwent systematic screening and prioritization. Compounds with docking scores over 75, lacking PAINS scaffolds, and interacting at the XPF protein’s Lys860 or adjacent residues were retained for further investigation. Visual assessment was pivotal in finalizing the selection, resulting in 48 compounds, which were then clustered by structures. Within the DrugBank compound list, five clusters emerged. Cluster 1, presenting the prevalent scaffold, was the most populated, with six structures (see Figure 4). This analysis pinpointed different substituents on a shared scaffold. Hence, only Bisoprolol and Betaxolol were selected for further examination due to their docking pose within the pocket and superior scores within this group. After comprehensive data analysis, seven DrugBank compounds were selected for biological evaluation, i.e. Terfenadine (DB1), Travoprost (DB2), Bisoprolol (DB3), Gadoteridol (DB4), Betaxolol (DB5), Ibutilide (DB6), and Reboxetine (DB7). A similar procedure was applied to assess the NCI and ChemBridge dataset results (see Figure 5 for schematic representation).

In the context of the NCI database, 284,176 compounds underwent preliminary screening. The top 5000 compounds exhibiting the highest scores were shortlisted for further analysis. These compounds were further assessed, commencing with a filtration step where only those with a score exceeding 90 were retained; this threshold matches the score of the best inhibitors from the ChEMBL dataset, see Figure 5. Ultimately, a set of 328 compounds were visually inspected, leading to the selection of a final set of 95 compounds for acquisition. Of these 95 compounds, only 35 were available through the NCI platform and were subsequently ordered for experimental testing. In this dataset, 13 clusters were identified. Both clusters 1 and 2 contained 10 molecules each. One important note is that the most representative scaffold determined via the Tanimoto coefficient may not always be relevant for the comparative analysis of the compounds, especially when other groups besides the shared scaffold differ significantly. Cluster 3 identified a secondary amide substituted with two alkyl chains. Upon examination of all structures within this cluster, substantial diversity among the nine molecules was observed. Consequently, relying solely on this analysis to guide compound selection may not be feasible.

For the ChemBridge database, a total of 1,008,227 compounds were screened using a workflow similar to the one applied for the NCI database. The top 5000 compounds with the highest scores were kept. In this dataset, 25 clusters were identified, with cluster 1 comprising 30 molecules sharing the same scaffold. Molecules from each cluster underwent visual inspection in relation to their pocket placement, guiding compound selection accordingly. All chosen molecules presented scores above 90. A final subset of 64 compounds was identified and subsequently purchased.

In summary, throughout the three virtual screening campaigns targeting the XPF protein, a total of 106 compounds were selected for subsequent biological evaluation. These were sourced from the NCI (35 compounds), ChemBridge (64 compounds), and DrugBank (7 compounds) databases.

**Figure 6:**
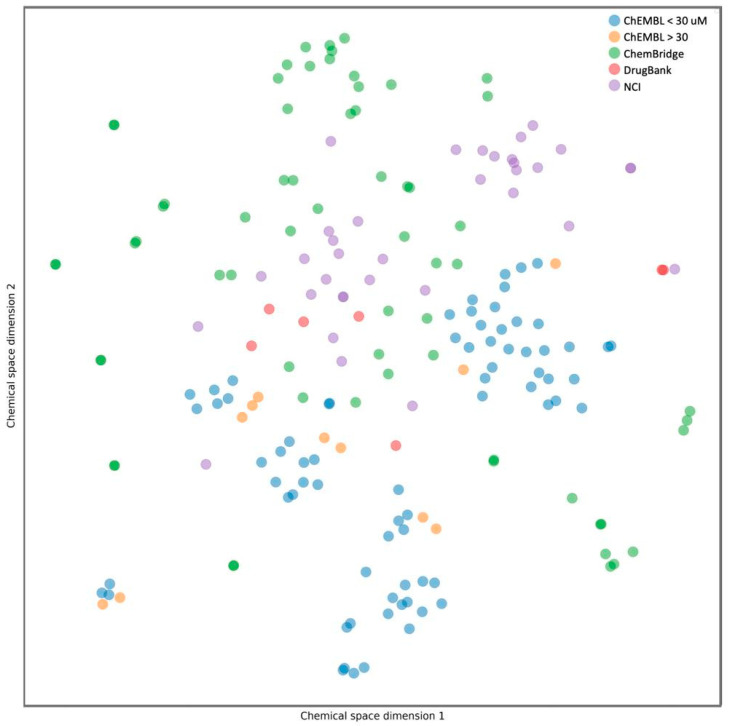
t-SNE distribution representing the chemical diversity of active (depicted in blue) and inactive (depicted in orange) compounds from ChEMBL. Additionally, the selected group of molecules from the NCI, Chembridge, and DrugBank databases are represented in purple, green, and red, respectively. The active compounds exhibit a broader chemical diversity than their inactive counterparts.

One of the primary objectives of this drug design protocol was to identify compounds with diverse chemical profiles for evaluation. To assess this diversity, the chemical space [59] of each final dataset of the selected compounds was mapped, as illustrated in Figure 6. This plot depicts the chemical space of the compounds from ChEMBL with activities either greater or lesser than 30 nM, alongside those selected from the NCI, Chembridge, and DrugBank. Notably, there is a discernible dispersion of compounds when comparing those selected from the virtual screening to those from ChEMBL. Compounds from ChEMBL cluster more closely together, predominantly because their structures are often modifications of the F06 inhibitor. Conversely, the virtual screening campaigns described here employed structures without any specific structural constraints tied to this inhibitor or other active compounds.

**Figure 7:**
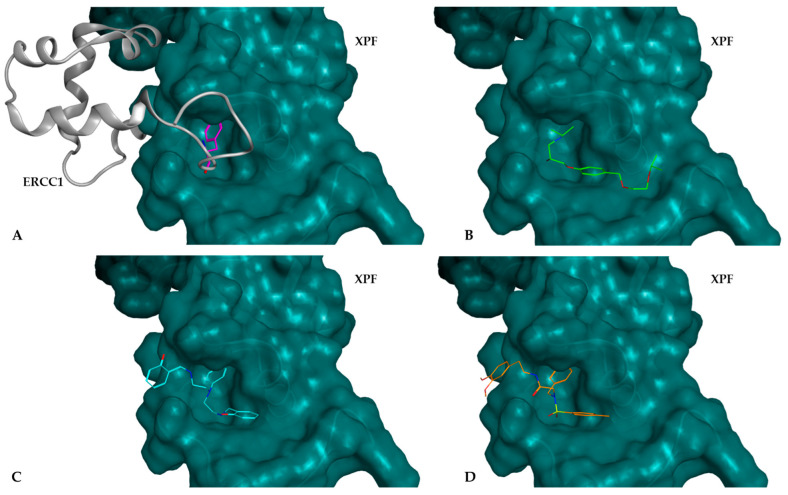
Representation of compounds docked into the XPF protein binding pocket: (**A**) ERCC1–XPF complex, highlighting the Phe293 residue (shown in pink) from ERCC1 located inside the binding pocket; Docked complexes of XPF with potential inhibitor selected from (**B**) DrugBank (Bisoprolol—identified as DB3—shown in green), (**C**) the NCI database (identified as NCI35—shown in blue), and (**D**) the ChemBridge database (identified as CB2—shown in orange). All these molecules fit snugly within the pocket, effectively obstructing its access.

In Figure 7, the potential inhibitors selected from each database are compared with the position of Phe293 residue from ERCC1 within the XPF pocket. Both compounds and the residue participate in a molecular interaction with the Lys860 residue from the XPF protein. The compounds from NCI (labeled C, colored blue) and ChemBridge (labeled D, colored orange) orient their aromatic moieties towards the upper part of the pocket, effectively occupying the entrance to the pocket. Conversely, the compound from DrugBank, Bisoprolol (labeled B, colored green), positions its primary alkyl primary amine group in the same region as the aromatic rings of the other compounds. It is noteworthy that the Phe293 residue also displays an aromatic side chain in this same region.

### 2.2. Characterization of NSCLC Cell Lines

#### 2.2.1. *ERCC1* and *ERCC4* Gene Expression in NSCLC Cell Lines

To evaluate the basal levels of ERCC1–XPF, attributing an NER status to each NSCLC cell line, mRNA levels of the desired genes were quantified using qRT-PCR. The *ERCC1* gene translates into the ERCC1 protein, forming a complex with the XPF endonuclease (coded by the *ERCC4* gene).

**Figure 8:**
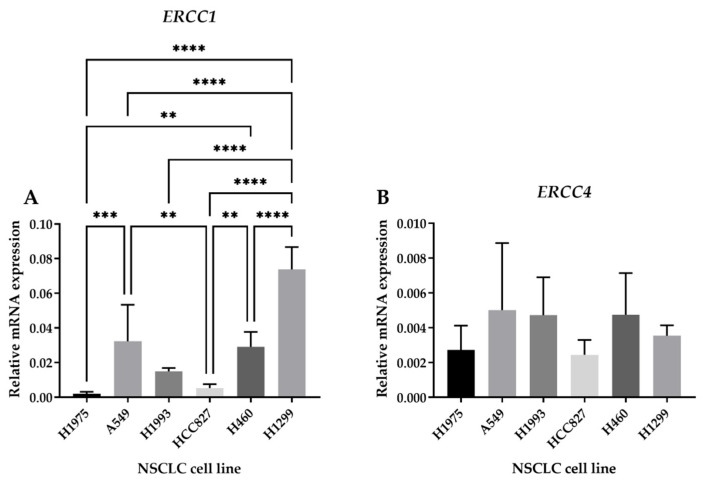
Relative mRNA expression levels (*β*-*Actin*) of genes *ERCC1* and *ERCC4* for all the NSCLC studied cell lines. Total RNA was isolated from six NSCLC cell lines and gene expression was analyzed using qRT-PCR for (**A**) *ERCC1* and (**B**) *ERCC4* genes. Relative expression of the two genes in each cell line was calculated using the expression 2^−∆Ct^, where ∆Ct = Average Ct (gene of interest) − Average Ct (*β*-*Actin*) and was normalized to the reference gene *β-Actin*. The results are expressed as mean ± SD (n = 3). ** *p* < 0.01, *** *p* < 0.001, and **** *p* < 0.0001 (one-way ANOVA, Tukey’s multiple comparisons test).

From all the studied cell lines, H1299 presented the highest *ERCC1* expression (Figure 8A). This cell line was followed by A549 cells in terms of *ERCC1* expression. Moreover, for *ERCC1* expression, H1975, HCC827, and H1993 cells presented the lowest value. There was no statistical significance between all cell lines regarding *ERCC4* expression (Figure 8B).

The results for *ERCC1* expression are in accordance with those obtained by Cai et al. [60]. These authors also observed a higher expression for H1299 cells, followed by the A549 cell line. It was also reported that H1975 and HCC827 cells presented the lowest levels of *ERCC1*, being in the same range. Cheng et al. [61] also obtained the same results regarding A549 and HCC827 cells, with A549 cells having the highest expression. Relevant to our results showing that H1299 cells have the highest expression of *ERCC1*, He et al. also corroborated this high expression pattern [62]. Altogether, the H1299 cell line was selected for the following cell-based assays.

#### 2.2.2. Cisplatin Cytotoxicity Profile in H1299 Cells

**Figure 9:**
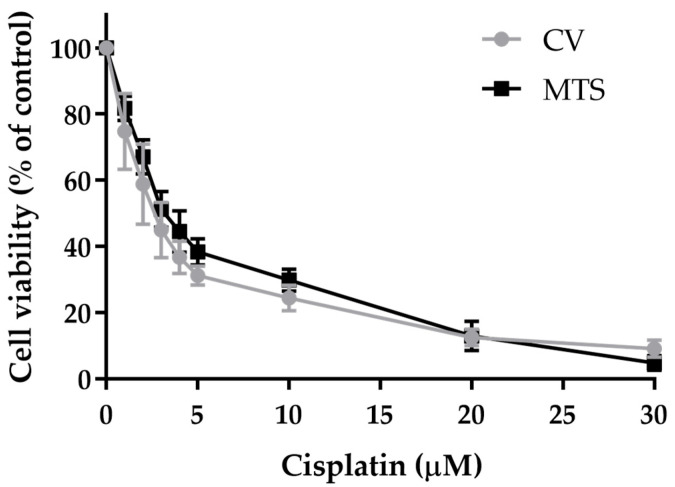
Cytotoxic effects of cisplatin in H1299 cells. The viability of cells treated with cisplatin for 72 h was assessed using the CV staining assay and the MTS reduction assay (n = 2–5). Values represent mean ± SD and are expressed as percentages of the vehicle-treated control cells.

To evaluate the cisplatin cytotoxicity effect in the selected H1299 cell line (highest *ERCC1* expression), a concentration–response profile was established using two mechanistically different cell viability assays, the Crystal Violet (CV) staining and the MTS reduction assays (Figure 9). The IC_50_ values for cisplatin were also calculated for a 72-h incubation period based on its dose–response profile.

Cell viability decreased in a concentration-dependent manner and the profiles were similar for both assays. Consequently, H1299 cells presented IC_50_ values of 3.0 µM and 3.7 µM based on the results from the CV and MTS assays, respectively.

### 2.3. Validation of the Putative ERCC1–XPF Inhibitors in In Vitro Cell-Based Assays

#### 2.3.1. Screening of the Putative ERCC1–XPF Inhibitors in Combination with Cisplatin on H1299 Cells

The next step of this work was to verify if the selected inhibitors do indeed enhance the cytotoxicity induced by cisplatin. With this objective in mind, an MTS assay was performed as a measure of cell viability. As already mentioned, H1299 cells were selected for these assays since they displayed the highest expression levels of ERCC1, being thus adequate as a model to mimic a clinical situation present in a subset of patients whose tumors overexpress this NER component. In short, cells were incubated with both cisplatin (1 µM) and each putative inhibitor (50 µM) for 72 h.

The concentration of 1 µM of cisplatin was selected since it encompasses a decrease in terms of cell viability of about 10–15%. Albeit not marked, this level of cytotoxicity enabled us to clearly identify the enhancement of the cytotoxic effects promoted by the putative inhibitor upon combination with cisplatin. As for the putative inhibitors, the concentration of 50 µM was selected since it represents, in our opinion, a balanced compromise between two major aspects that should be considered, i.e., efficacy as NER inhibitors vs. inherent compound toxicity. In fact, if higher concentrations were selected (e.g., 100 µM), it would be plausible that a larger number of compounds could be revealed as cytotoxic per se due to their inherent toxicological properties. On the other hand, if we decrease this concentration level at the initial screening phase, we might lose a number of interesting compounds that function only at higher concentrations.

**Figure 10:**
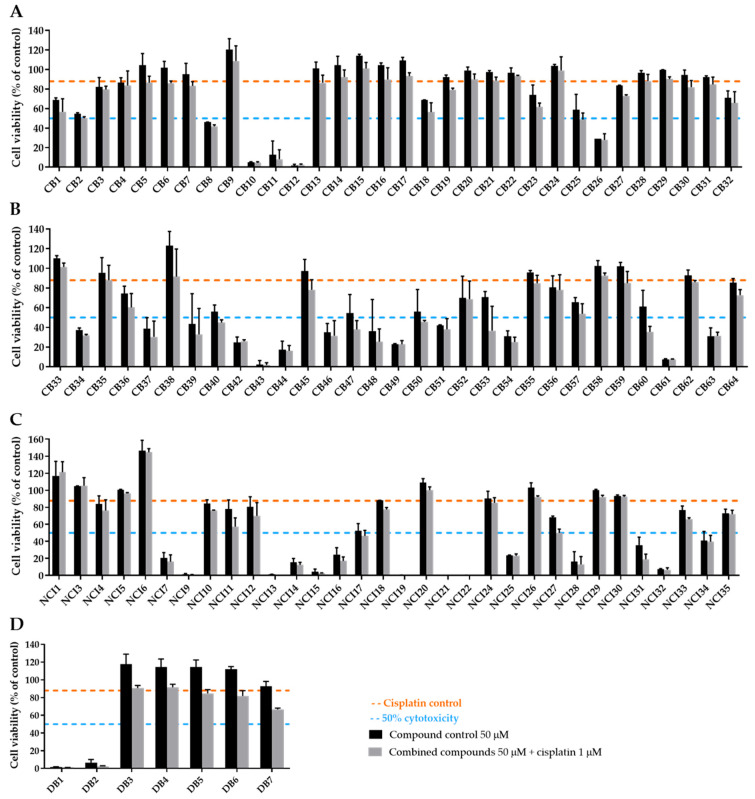
Assessment of the sensitizing effect of the putative ERCC1–XPF inhibitors in terms of cisplatin-induced cytotoxicity. An MTS assay was performed, and all compounds (50 µM) selected from (**A**,**B**) ChemBridge, (**C**) NCI, and (**D**) DrugBank databases were incubated with cisplatin (1 µM) for 72 h. Values represent mean ± SD and are expressed as percentages of the vehicle-treated control cells considered as 100% of cell viability (n = 2–3).

The results for all compounds selected from the ChemBridge, NCI, and DrugBank databases are depicted in Figure 10. However, one compound (CB41) of the 64 compounds ordered from ChemBridge was not available, and three compounds (NCI2, NCI8, and NCI23) from NCI were not soluble in DMSO, precluding its evaluation in the cell-based assays. For this reason, a total of 102 molecules were evaluated.

**Figure 11:**
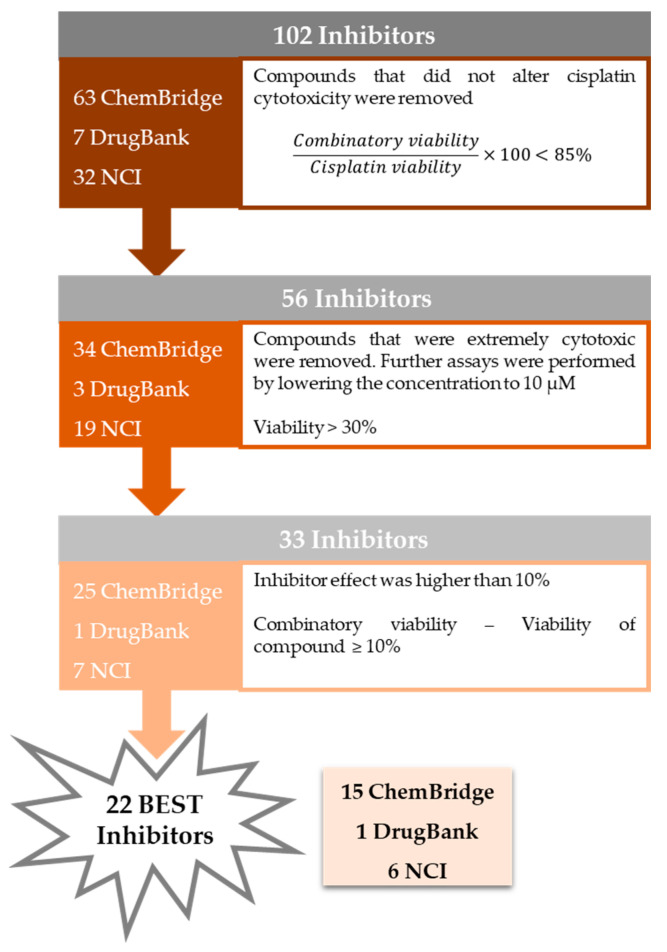
Decision-making process employed to achieve the 22 BEST inhibitors.

**Figure 12:**
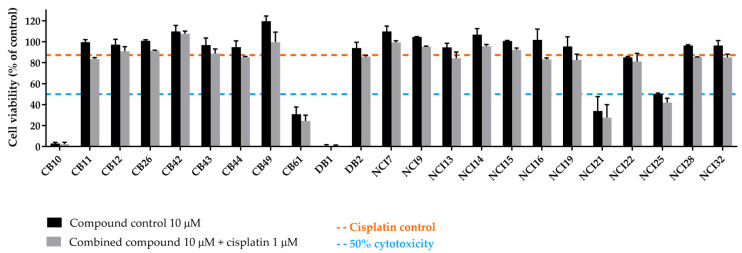
Evaluation of the sensitizing effect of the ERCC1–XPF inhibitors, which were considered extremely cytotoxic at 50 µM, in cisplatin-induced cytotoxicity. An MTS assay was performed in H1299 cells, and all compounds (10 µM) were incubated with cisplatin (1 µM) for 72 h. Values represent mean ± SD and are expressed as percentages of the vehicle-treated control cells considered as 100% of cell viability (n = 2).

With the purpose of selecting the best inhibitors among the pool of 102 tested compounds, a decision-making process was systematically employed. This strategy is depicted in Figure 11. In short, the first step was to evaluate the impact of the combination of cisplatin with the putative inhibitor in comparison with cisplatin alone in H1299 cells. To achieve this, the cell viability presented by the combined condition relative to cisplatin viability was calculated, and, if lower than 85%, the compounds moved to the next stage. The use of this threshold identifies compounds that increase cisplatin cytotoxicity by at least 15%. From this step, 46 compounds were discarded (i.e., 29 from CB, 4 from DB, and 13 from NCI). The next step addressed the fact that some putative inhibitors are indeed highly cytotoxic per se. In fact, while some of them completely abolished cisplatin viability upon combination, some were concomitantly toxic alone. Therefore, if the compounds alone at 50 µM induced more than 70% cytotoxicity (i.e., presented viability < 30%), they were removed at this stage from the pool of best inhibitors. Nevertheless, these compounds were not yet discarded but rather moved to a next approach that consists of lowering the concentration tested in cells to 10 µM (five-fold; for these results, see Figure 12). As a result, 23 compounds were taken out of the pool of the top compounds (i.e., 9 from CB, 2 from DB, and 12 from NCI). From the remaining 33 putative inhibitors, a next decision step was introduced to consider the influence directly ascribed to the putative inhibitor in the combinatory effect and not merely the result of its own cytotoxicity in NSCLC cells. To verify this, the difference between the viability of the inhibitor alone was subtracted from the combinatory condition. This information allowed the calculation of the inhibitor effect, and, if it was higher than 10%, it was considered in the short list of the best inhibitors. The resulting BEST inhibitors were 15 from ChemBridge, 1 from DrugBank, and 6 from the NCI databases (total of 22 compounds, i.e., CB1, CB18, CB23, CB25, CB27, CB36, CB39, CB40, CB47, CB48, CB50, CB53, CB57, CB60, CB64, DB7, NCI11, NCI12, NCI18, NCI27, NCI31, and NCI33), to be used in further studies. The chemical structures of the 22 best inhibitors are present in Supplementary Materials, Figure S1.

#### 2.3.2. Extremely Cytotoxic Compounds in H1299 Cells

Since some compounds were demonstrated to be extremely cytotoxic to NSCLC cells (cell viability less than 30%), a further step was performed by lowering the concentration applied to the cells (Figure 12). In this additional assay, a concentration of 10 µM was selected for each compound to be combined with 1 µM of cisplatin, and a total of 23 compounds were tested (9 from ChemBridge, 2 from DrugBank, and 12 from NCI databases).

Even with the strategy of reducing the concentration of the extremely cytotoxic compounds to 10 µM, there were still five compounds that induced more than 50% of cell death, i.e., CB10, CB61, DB1, NCI21, and NCI25. Nevertheless, none of the other 18 compounds could enhance cisplatin cytotoxicity in a significant manner. For this reason, they were not selected for further studies.

### 2.4. Interaction Analysis of Inhibitors with XPF Protein

**Figure 13:**
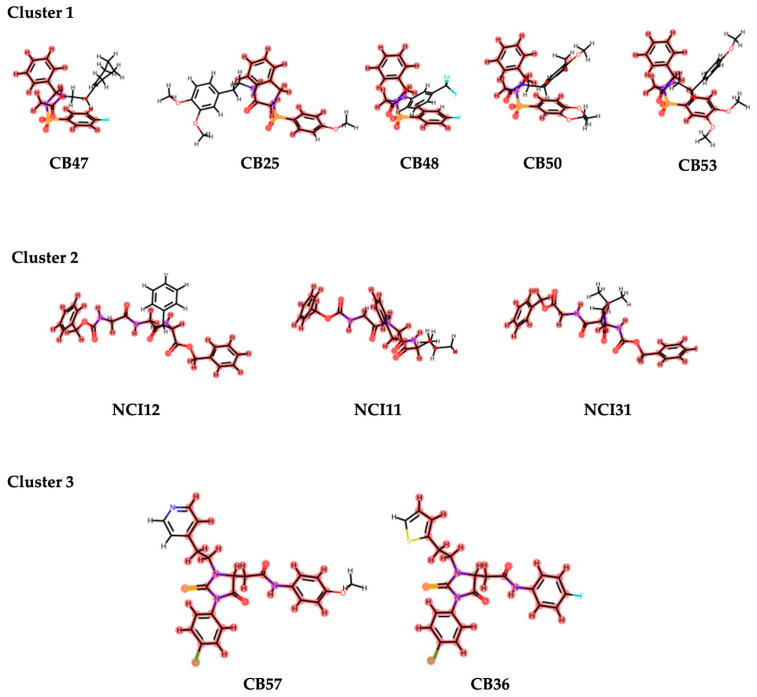
Representation of clusters derived from the final data set of 22 molecules proposed as inhibitors. Between the 22 molecules, 3 clusters were identified, and cluster 1 had the highest number of molecules. Cluster 2 presented 3 molecules, and cluster 3 had 2 molecules. Parts of the molecules highlighted in red represent the similar scaffold that defines each cluster.

**Figure 14:**
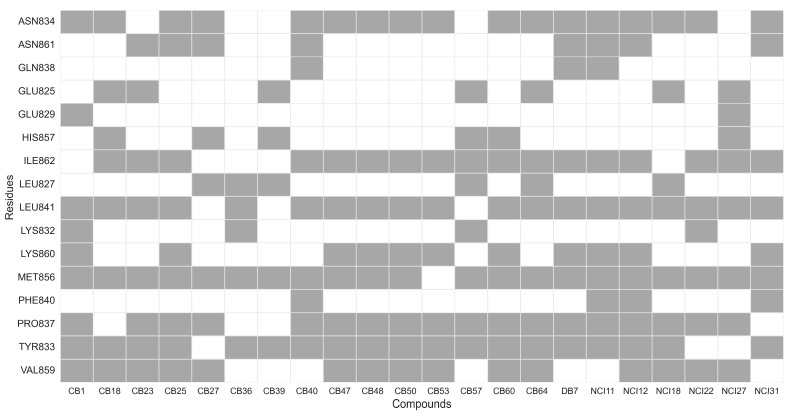
Heatmap representation of XPF residues involved in interactions with the best 22 compounds: each row represents specific XPF residues, while each column corresponds to a compound. Squares in gray indicate residues where ligand–receptor interactions occur, while the white squares represent the absence of such interactions.

**Figure 15:**
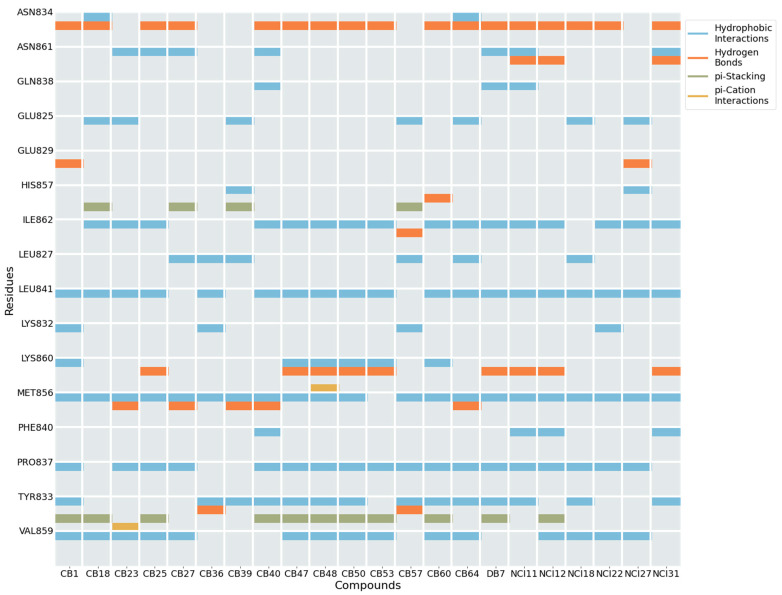
Representation of the types of interactions established between the XPF protein and the tested compounds. Each row represents the XPF residues, while the columns identify each one of the 22 molecules. Our analysis considered various interactions, including H-bonds, hydrophobic interactions, π-stacking, water bridges, halogen bonds, and salt bridges. Water bridges and π-stacking interactions between these molecules and residues were not identified.

Upon identifying the top 22 inhibitors from a pool of 102 tested compounds, a detailed structural analysis was conducted to elucidate the interaction mechanisms between these molecules and the XPF protein. Within this selection, three distinct clusters were identified. Cluster 1, consisting of five molecules (CB25, CB47, CB48, CB50, CB53), exhibited a common structural scaffold, characterized by a sulfonyl group surrounded by three aromatic rings (Figure 13). The key molecular interactions between these compounds and the protein involved residues such as Asn834, Ile862, Leu841, Lys860, Met856, Pro837, Tyr833, and Val859 (Figure 14). The predominant types of interactions were hydrogen bonding, hydrophobic interactions, and π–cation interactions, which typically involve aromatic rings (Figure 15). Cluster 2, comprising three molecules from the NCI dataset (NCI11, NCI12, NCI31), shared a structural motif with two aromatic rings connected by an ester chain (Figure 13). This cluster exhibited hydrophobic interactions involving Phe840, along with interactions identified in Cluster 1. Cluster 3 included two molecules from the ChemBridge dataset, CB36 and CB57, which mainly differed in the type of aromatic ring present. Despite their structural similarities, these molecules showed the various residues involved in the molecular interactions (Figure 13).

The analysis of the protein residues involved in the interaction between the receptor and the ligand facilitated the identification of key residues that potentially promote XPF inhibition when interacting with the dataset of 22 molecules. Predominantly, hydrophobic interactions were observed between these 22 compounds and the XPF residues. A significant occurrence of hydrogen bonds and aromatic interactions, including π−π stacking and π-cation interactions, was noted. Residues Tyr833, Pro837, Leu841, and Met856 were found to interact with the highest number of compounds, suggesting their crucial role in potentially promoting XPF inhibition. These residues predominantly engage in hydrophobic interactions, as depicted in Figure 15.

## 3. Materials and Methods

### 3.1. Computational Simulations

#### 3.1.1. Selection and Preparation of Protein Structures

The ERCC1–XPF endonucleases complex was identified as a potential target for NER inhibitors. As of the time of this study, eight three-dimensional structures of the ERCC1–XPF complex were accessible on the Protein Data Bank (PDB) (Table 1) [46]. Among these, none featured small-molecule inhibitors, and only one showed the complex interacting with DNA. All the crystal structures available represent the human variant of the complex.

For our analysis, we narrowed our focus to four structures that showcase the complex C-terminal domains of human XPF and ERCC1, aligning with our region of interest. These specific structures are denoted as 1Z00, 2A1J, 6SXA, and 6SXB. These complex structures were retrieved from the PDB database. The preparation of each crystal structure was undertaken using the Molecular Operating Environment (MOE v.2020.0901) structure preparation module [53]. Hydrogen atoms were added, and appropriate protonation states were assigned using the Protonate-3D tool at pH 7.4 within the MOE software package v2020.0901. Crystallographic water molecules and other non-relevant entities not associated with the ERCC1 and XPF structures were excised.

#### 3.1.2. Selection and Preparation of the Ligands

A comprehensive search of the ChEMBL database was conducted to retrieve a dataset of known small-molecule inhibitors targeting ERCC1–XPF [56]. As of December 2021, 84 entries were identified. The SMILES notation of these compounds was procured and subsequently converted into 3D structures. Each molecule was prepared using the Protonate 3D tool within MOE v.2020.0901, at pH 7.4 and 300 K. Subsequently, the molecules underwent energy minimization using the PM7 semi-empirical method to ensure an optimal starting conformation. The ligands’ physicochemical properties were assessed using both MOE software [53] and the FAF-Drugs4 webserver [57].

#### 3.1.3. Validation of Docking and Virtual Screening Approaches

To validate our docking methodology, benchmark studies were carried out using the GOLD v5.07 software package. We assessed four scoring functions: ChemPLP [63], GoldScore [64], ChemScore [65], and ASP [66]. Each identified small molecule, termed as a ligand, was docked onto the available protein crystal structures. We explored three different receptor configurations: the complete ERCC1–XPF complex and XPF or ERCC1 proteins alone. Independent docking simulations were performed for each ligand, scoring function, and receptor combination.

The docking site was anchored using Lys860 residue and Ile862 residues, both crucial for complex integrity, with a 15 Å radius around them. All scoring functions were assessed for their efficacy in scoring and ranking. Based on our evaluation, ChemPLP was chosen for subsequent virtual screening efforts, employing 500 GA runs. We targeted three databases for further virtual screening: NCI [67], ChemBridge [68], and DrugBank [69]. National Cancer Institute (NCI) Library: This library, housing 284,176 compounds, was established and is curated by the Developmental Therapeutics Program of the National Cancer Institute at the National Institutes of Health (Bethesda, MD, USA). The selected compounds were generously provided by the institution, with researchers bearing only shipping expenses. ChemBridge Database: This commercial database encompasses roughly 2 million compounds. We purchased the selected compounds from this collection for our research. DrugBank Database: comprising approved and commercialized drugs, this database presents an opportunity for drug repurposing. With a total of 524 compounds having successfully completed clinical trials, there is an enhanced possibility of these molecules possessing favorable pharmacokinetic profiles.

Ranking and assessment of compounds from virtual screening: Docked-based virtual screening produced compounds that were ranked considering multiple criteria, such as scoring metrics, ligand efficiency, binding pocket fit, and interactions with adjacent residues. We conducted a thorough analysis of each compound, focusing on parameters like molecular weight, hydrogen-bond donors, hydrogen-bond acceptors, rotatable bonds, and logP values.

Compounds targeting the Lys860 residue in XPF were visually inspected using the MOE v.2020.0901 software. Emphasis for top-ranking molecules was on their position within the binding pocket. Compounds exhibiting potential PAINS interactions were systematically eliminated.

#### 3.1.4. Determination of Key interactions, Clustering Structurally Similar Scaffolds, and Chemical Space

We employed clustering to group together structurally similar scaffolds using the Murcko Scaffold decomposition method, which is available in RDKit [58]. The similarity between these scaffolds was gauged by calculating the Tanimoto coefficient, specifically utilizing Morgan fingerprints, also implemented in the RDKit. The RDKit was also used to calculate 2D and 3D physicochemical descriptors. Visual clustering was performed with t-SNE [59] dimensionality reduction, using the function implemented in scikit-learn (TSNE function), projecting the original 1024 dimensions into two final dimensions.

The PDB files for each studied system were processed using the Protein–Ligand Interaction Profiler (PLIP) algorithm through the PLIP Python module [70]. This tool examines and visualizes protein–ligand interactions in PDB files, facilitating a comparative assessment of interactions across systems. Our analysis considered various interactions, including H-bonds, hydrophobic interactions, π-stacking, water bridges, halogen bonds, and salt bridges.

### 3.2. Cell-Based Assays

#### 3.2.1. Chemicals

RPMI-1640 with L-glutamine and HEPES were purchased from Biowest (Nuaillé, France). Cisplatin and crystal violet (CV) were purchased from Sigma–Aldrich (Madrid, Spain). Penicillin–streptomycin solution (10,000 units/mL of penicillin; 10 mg/mL of streptomycin), trypsin (0.25%), and Fetal Bovine Serum (FBS) were acquired from Gibco (Paisley, UK). Dimethylsulfoxide (DMSO) was obtained from Fisher Chemical (Waltham, MA, USA). Sodium pyruvate was purchased from PAN Biotech (Aidenbach, Germany). D-glucose and sodium bicarbonate were purchased from AppliChem (Darmstadt, Germany). Sodium chloride was obtained from Labchem (Lisboa, Portugal).

A 2 mM stock solution of cisplatin was prepared in saline (0.9% NaCl), aliquoted, and stored at −20 °C. Further diluted stock solutions were also prepared in saline, aliquoted, and stored at −20 °C.

The acquired compounds from the Chembridge, NCI, and DrugBank databases were reconstituted in 100% DMSO at a concentration of 12.5 mM, aliquoted, and stored at −20 °C. A further dilution to 2.5 mM was also performed in 100% DMSO and stored at −20 °C when needed. Working solutions of each compound were freshly diluted in a complete cell culture medium so that, in each final solution, the DMSO concentration was kept at 0.4% (*v*/*v*) when in contact with cells.

In all cell-based assays, vehicle-treated controls were also included, in which cells were exposed to the respective solvents, i.e., DMSO (final concentration of 0.4% (*v*/*v*)) and/or saline.

#### 3.2.2. Cell Culture

The human NSCLC H1975, A549, H460, H1993, HCC827, and H1299 cell lines were obtained at the American Type Culture Collection (ATCC, Manassas, VA, USA). All cell lines were cultured in monolayer in RPMI-1640 medium with L-glutamine supplemented with 10 mM HEPES, 2.5 g/L D-glucose, 1 mM Sodium pyruvate, 1.5 g/L Sodium bicarbonate, 10% FBS, and 1% Pen/Strep (complete cell culture medium) and were maintained at 37 °C under a humidified atmosphere containing 5% CO_2_ in the air.

#### 3.2.3. Gene Expression

To select the best NSCLC cell model to study the putative inhibitors, a characterization of the expression of the *ERCC1* and *ERCC4* (XPF) genes in all cell lines was performed by using quantitative reverse transcription PCR (qRT-PCR), following a previously described protocol [71]. Total RNA was isolated from each cell line (1.0 × 10^6^ cells/sample) using TRIzol™ Reagent (Invitrogen™, Waltham, MA, USA) and extracted according to the manufacturer’s instructions. RNA concentration was assessed by measuring the absorbance at 260 nm using the LVis plate mode (SPECTROstar Omega, BMG Labtech, Ortenberg, Germany). cDNA was synthesized from 1 μg of RNA using an NZY First-Strand cDNA Synthesis Kit (NZYTech, Lisbon, Portugal) according to the manufacturer’s instructions. qRT-PCR was performed using PowerUp™ SYBR^®^ Green Master Mix (Applied Biosystems™, Waltham, MA, USA) for a final reaction volume of 15 μL, using 2 μL of template cDNA and 0.33 μM of forward and reverse primers. The primer sequences used were the following: *ERCC1*, forward, 5′-CTACGCCGAATATGCCATCTC-3′, reverse, 5′-GTACGGGATTGCCCCTCTG-3′; *ERCC4* (XPF), forward, 5′-CACCTCCCTCGCCGTGTA-3′, reverse, 5′-CGCAAATATAACACCACCTTGTG-3′; *β*-*Actin*, forward, 5′-CATGTACGTTGCTATCCAGGC-3′, reverse, 5′-CTCCTTAATGTCACGCACGAT-3′. The reaction was performed on a QuantStudio™ 7 Flex Real-Time PCR System (Applied Biosystems, Waltham, MA, USA) and consisted of a 2-min uracil-N-glycosylase activation step at 50 °C and a denaturation step at 95 °C for 10 min, followed by 40 cycles of a denaturation step of 15 s at 95 °C and annealing/extension at 60 °C for 1 min. Finally, a dissociation step was added to determine the melting temperature of a single target sequence as a measure of quality and specificity. Relative expression of the two target genes in each cell line was calculated using the expression 2^−∆Ct^, where ∆Ct = Average Ct (gene of interest)—Average Ct (*β*-*Actin*), and it was normalized to the reference gene *β*-*Actin* [72]. qRT-PCR reactions were performed on duplicate samples of cDNAs from three independent collections of all cell lines.

#### 3.2.4. Crystal Violet Staining Assay

The cytotoxicity of cisplatin in H1299 cells was evaluated according to a previously described crystal violet (CV) staining protocol [73]. This assay allows the colorimetric determination of adherent cells by staining their nucleus. First, cells were inoculated at a density of approximately 3 × 10^3^ cells/well in 200 μL of complete culture medium in 96-well plates. After adherence (24 h), the culture medium was renewed, and the cells were incubated with a range of 1–30 µM of cisplatin for another 72 h. After incubation, cells were washed with warm PBS to remove non-adherent cells (non-viable cells). The adherent cells were then fixated with ice-cold 96% ethanol for 10 min and stained with 0.1% crystal violet in 10% ethanol for 5 min. The plate was then washed with tap water and the stained cells were dissolved in 200 µL of 96% ethanol with 1% acetic acid. Absorbance was measured at 595 nm (OD595) using a SPECTROstar OMEGA microplate reader. Values presented by vehicle-treated control cells (untreated cells) corresponded to 100% of cell viability. Three to five independent experiments were performed with six replicates for each condition in each independent experiment. The IC_50_ values were calculated based on the obtained concentration–response curves using GraphPad Prism^®^ 9.0 (GraphPad Prism^®^ 9.0 Software, Inc., La Jolla, CA, USA).

#### 3.2.5. MTS Reduction Assay

The 3-(4,5-dimethylthiazol-2-yl)-5-(3-carboxymethoxyphenyl)-2-(4-sulfophenyl)-2H-tetrazolium salt (MTS) reduction assay was carried out as a confirmatory assay of cell viability. This methodology is commonly used in in vitro toxicology and is based on the enzymatic reduction of a tetrazolium salt in viable cells by mitochondrial enzymes (NAD-dependent dehydrogenase) with the formation of a colored formazan product. Briefly, after incubation with the compound and removal of the complete cell culture medium, cells were washed with warm PBS, followed by the addition of 100 μL of fresh medium and 20 μL of MTS substrate prepared from the CellTiter 96^®^ Aqueous MTS (Promega, Madison, WI, USA) according to the manufacturer’s instructions. H1299 cells were incubated for 2 h 30 with the MTS reagent, and the absorbance was measured at 490 nm and 690 nm (reference wavelength) using a SPECTROstar OMEGA microplate reader. Absorbance values presented by vehicle-treated control cells corresponded to 100% of cell viability. Two to four independent experiments were performed, and three replicates were used for each condition in each independent experiment. The IC_50_ was also calculated based on the concentration–response curve using GraphPad Prism^®^ 9.0.

#### 3.2.6. First Screening Approach of the ERCC1–XPF Inhibitors in an NSCLC Cell Line

The compounds previously identified as putative ERCC1–XPF inhibitors were first evaluated in a set of combinatory cell-based experiments with cisplatin. A concentration of 50 µM of each compound was combined with 1 µM of cisplatin and applied to the H1299 cells (highest *ERCC1* expression). The MTS reduction assay was performed, as previously described, for the assessment of cell viability, and a 72-h incubation period was conducted for both compounds. For the compounds that were considered to be highly cytotoxic in the first approach, a further assay was performed in the same cell line by lowering their concentration to 10 µM. Two to three independent experiments were performed.

#### 3.2.7. Statistical Analysis

The results are presented as average ± SD. Data were analyzed with one-way ANOVA multiple comparisons with GraphPad Prism^®^ 9.0. *p* < 0.05 was considered statistically significant (represented as: * *p* < 0.05, ** *p* < 0.01, *** *p* < 0.001, and **** *p* < 0.0001).

## 4. Conclusions

Components of the NER pathway have emerged as druggable targets, namely, the ERCC1–XPF complex, with important implications in NSCLC therapy. Different studies have suggested that interfering with the complex function could improve standard platinum-based chemotherapy. For this reason, we proposed the development of novel ERCC1–XPF inhibitors that could enhance cisplatin efficacy in NSCLC patients.

In the present work, we were able to identify novel molecules for ERCC1–XPF inhibition. Namely, a successful SBVS protocol was implemented, the previously reported inhibitors were retrieved from ChEMBL, the molecular docking calculations were concluded, and a thorough virtual screening campaign led to the identification of a pool of novel putative inhibitors for acquisition in different databases (ChemBridge, NCI, and DrugBank). The selection of a given compound to proceed in further biological tests was based on a combination of general performance, considering the score, the molecular weight, lipophilicity, ligand efficiency, and the appropriate pocket fitting and interactions with the nearby residues. The visual inspection was a very important step in this assessment due to the ability to understand the compounds fitting into the pocket and interaction with the ligand. With these implemented strategies, we were able to propose 106 compounds for acquisition to be used in further analysis.

**Figure 16:**
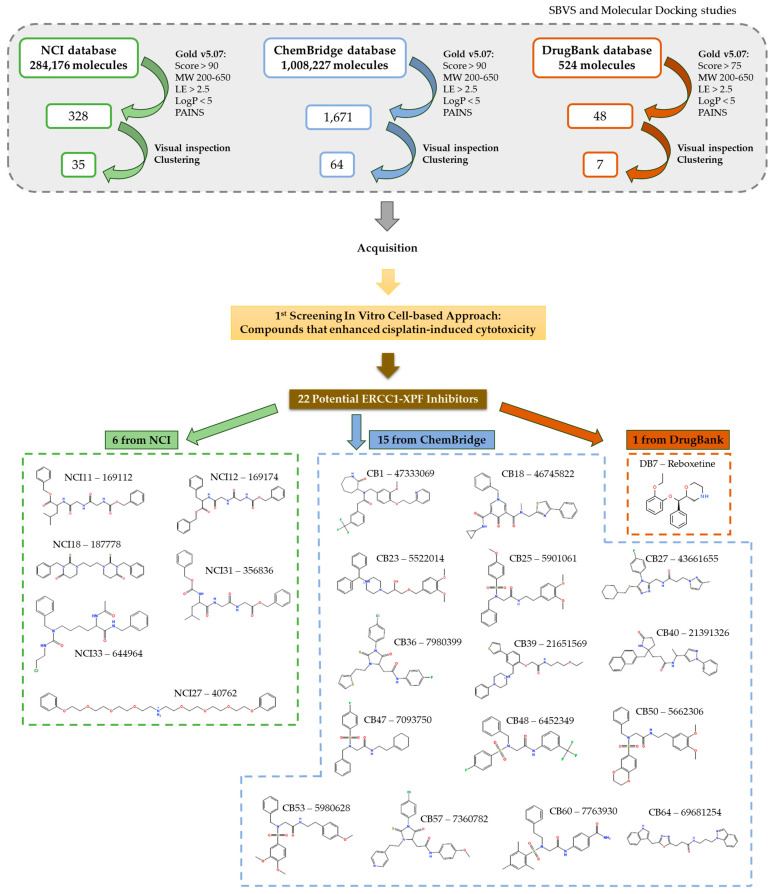
Overview of the protocol employed for the identification of the 22 best ERCC1–XPF inhibitors.

Keeping in mind that the main goal of this work was to find novel active inhibitors to enhance cisplatin activity, an initial screening approach was developed in vitro. This approach comprised the identification of compounds that could, in fact, enhance cisplatin-induced cytotoxicity in NSCLC cell lines with high *ERCC1* expression. From all the tested 102 compounds, we were able to identify 22 compounds that demonstrated positive results in enhancing cisplatin-induced cytotoxicity. An overview of the protocol employed for this purpose is depicted in Figure 16. All these compounds are simple small molecules with good spatial fitting in the ERCC1–XPF active binding site and establish important interactions with residues essential for complex formation.

Overall, our findings illustrate the importance of addressing small-molecule key features to guide the discovery of modulators of ERCC1–XPF interaction, providing important insights leading to the identification of potential inhibitors with novel chemotypes to be used in combination with cisplatin therapy in NSCLC.

## Figures and Tables

**Table 1:** Compilation of experimental 3D structures available for the ERCC1–XPF complex in the Protein Data Bank (PDB). Structures shaded in gray were used for subsequent analyses.

PDB ID	Res. (Å)	Year	Complex	Ref.
6SXA	3.60	2020	ERCC1–XPF Cryo-EM structure, Apo-form	Jones et al., 2020 [47]
6SXB	7.90	2020	ERCC1–XPF Cryo-EM structure, DNA-bound form	Jones et al., 2020 [47]
2MUT	Solution	2015	Solution structure of the F231L mutant ERCC1–XPF dimerization region	Faridounnia et al., 2015 [48]
2KN7	Solution	2010	Structure of the XPF–single-strand DNA complex	Das et al., 2012 [50]
2AQ0	Solution	2006	Solution structure of the human homodimeric DNA repair protein XPF	Das et al., 2007 [51]
2A1J	2.70	2005	Crystal structure of the complex between the C-terminal domains of human XPF and ERCC1	Tsodikov et al., 2005 [49]
2A1I	1.90	2005	Crystal structure of the central domain of human ERCC1	Tsodikov et al., 2005 [49]
1Z00	Solution	2005	Solution structure of the C-terminal domain of ERCC1 complexed with the C-terminal domain of XPF	Tripsianes et al., 2005 [52]

## Data Availability

Data are contained within the article or Supplementary Materials.

## Supplementary Materials

The following supporting information can be downloaded at: https://www.mdpi.com/article/10.3390/ijms25021246/s1.
