# Supplementary material for: Unveiling Novel ERCC1–XPF Complex Inhibitors: Bridging the Gap from In Silico Exploration to Experimental Design
Source: Int J Mol Sci. 2024 Jan 19;25(2):1246. doi: 10.3390/ijms25021246 (PMC10816628; doi:10.3390/ijms25021246)
Supplement: Supplementary file 1 [file ijms-25-01246-s001.zip › ijms-2782320-supplementary.pdf]

Supplementary Materials

CB1—47333069 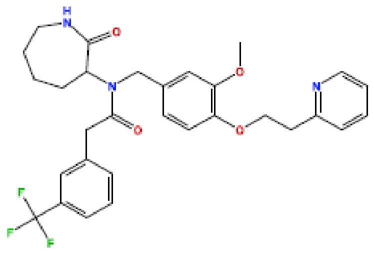	CB18—46745822 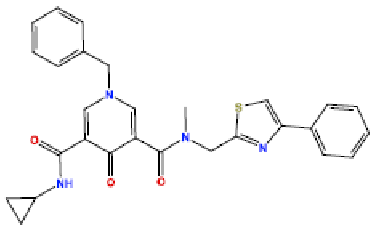	CB23—5522014 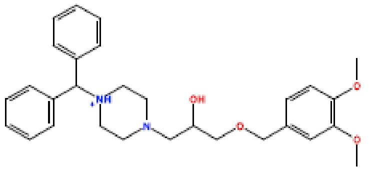
CB25—5901061 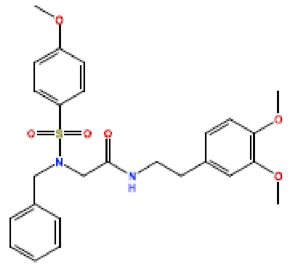	CB27—43661655 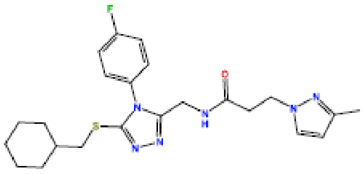	CB36—7980399 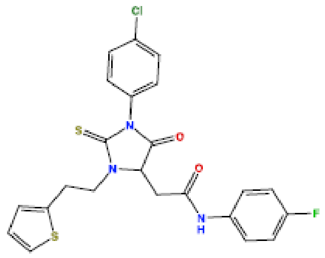
CB39—21651569 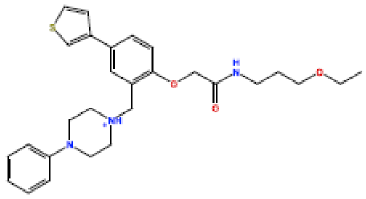	CB40—21391326 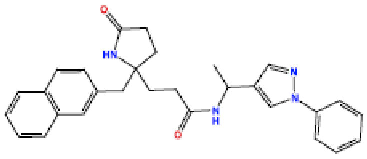	CB47—7093750 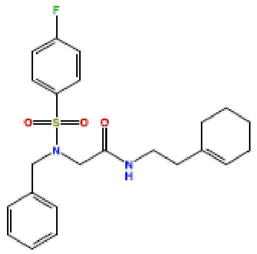
CB48—6452349 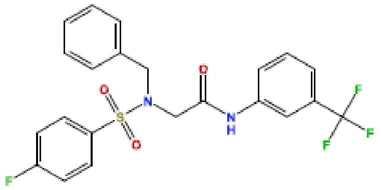	CB50—5662306 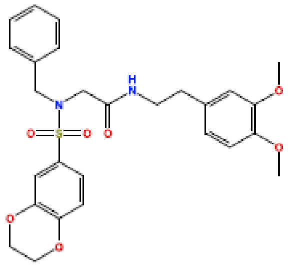	CB53—5980628 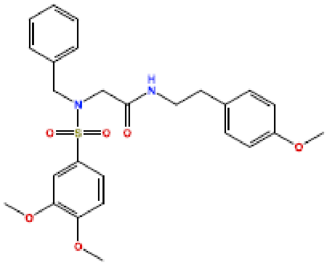
CB57—7360782 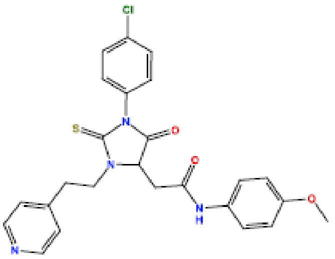	CB60—7763930 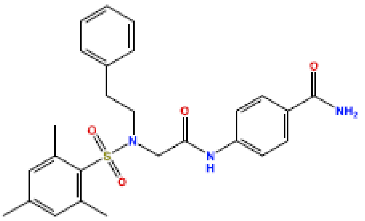	CB64—69681254 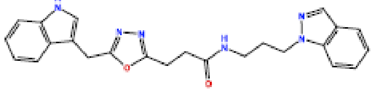
DB7—Reboxetine	NCI11—169112	NCI12—169174

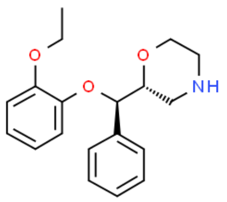	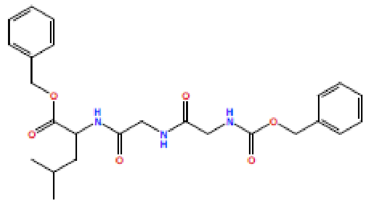	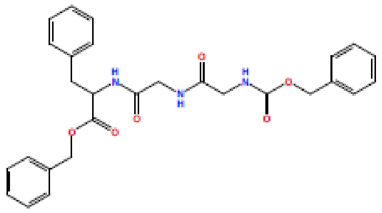
NCI18—187778 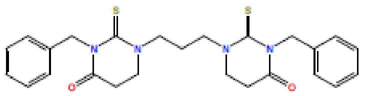	NCI31—356836 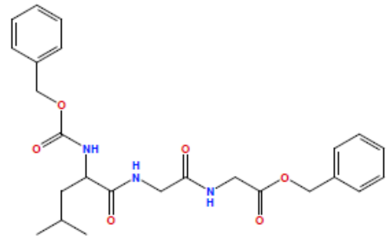	NCI33—644964 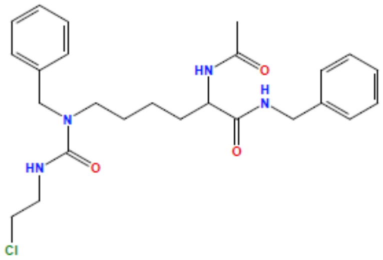
NCI27—40762 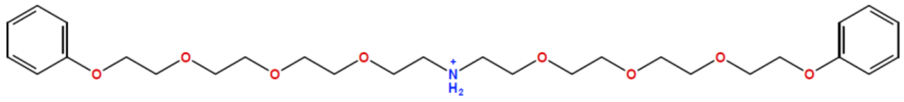		

Figure S1. List and chemical structures of the 22 compounds identified as best inhibitors that could enhance cisplatin-induced cytotoxicity.
